# Analysis of rhizosphere soil microbial diversity and its functions between Dahongpao mother tree and cutting Dahongpao

**DOI:** 10.3389/fpls.2024.1444436

**Published:** 2024-09-06

**Authors:** Xiaoli Jia, Mingzhe Li, Qi Zhang, Miao Jia, Lei Hong, Shuqi Zhang, Yuhua Wang, Yangxin Luo, Tingting Wang, Jianghua Ye, Haibin Wang

**Affiliations:** ^1^ College of Tea and Food, Wuyi University, Wuyishan, China; ^2^ College of Life Science, Longyan University, Longyan, China; ^3^ College of JunCao Science and Ecology, Fujian Agriculture and Forestry University, Fuzhou, China

**Keywords:** tea tree, microbial diversity, soil nutrient cycling, community structure, enzymatic activity

## Abstract

Dahongpao mother tree (*Camellia sinensis* (L.) O. Ktze) is a representative of Wuyi rock tea. Whether there is a difference in rhizosphere soil microbial diversity and function between asexually propagated cuttings of Dahongpao (PD) and the parent Dahongpao mother tree (MD) has not been reported. In this study, high throughput sequencing technology was used to analyze rhizosphere soil microbial diversity, functions and their relationship with soil available nutrients and enzyme activities in MD and PD. The results showed that available nitrogen, phosphorus and potassium contents and urease, protease, acid phosphatase and sucrase activities of rhizosphere soils in MD were significantly higher than those in PD. Both bacterial and fungal diversity were higher in rhizosphere soils in MD than in PD, and secondly, the bacterial community structure was less stable while the fungal community structure was more stable in PD compared to MD. There were significant differences between MD and PD tea tree rhizosphere soils in 6 genera of characteristic bacteria and 4 genera of characteristic fungi. The results of function and interaction effect analysis showed that the rhizosphere soil available nutrient content and enzyme activities in MD were significantly higher than those in PD, and their contributions mainly originated from *Pirellula* and *Acidisphaera* of characteristic bacteria and *Alatospora* of characteristic fungi. Secondly, MD maybe had a stronger ability to inhibit soil pathogens than PD, with the main contribution coming from *Scopulariopsis* and *Tolypocladium* of characteristic fungi. Overall, compared with PD, soil texture in MD was relatively better, and its soil nutrient cycling-related enzyme activities were stronger, which was more favorable to soil nutrient cycling and increased the available nutrient content of the soil, which in turn promoted the growth of tea trees. This study provides an important reference for the planting and management of tea tree cuttings and microbial regulation of tea tree growth.

## 1 Introduction

Dahongpao mother tree (*Camellia sinensis* (L.) O. Ktze) refers to the Dahongpao tea tree growing in Jiulongke Scenic Area of Wuyi Mountain, Fujian Province, with a total of six plants and an age of nearly 400 years (Chen, 2015). Dahongpao mother tree is the tea goddess of Wuyi Mountain and is the representative of Wuyi rock tea. In the 1930s, the local government sent soldiers to guard Dahongpao mother tree, and after the founding of the People’s Republic of China, the relevant departments still employ farmers to look after the tree year after year. In 2000, Wuyi Mountain was successfully declared a World Natural and Cultural Heritage site, and Dahongpao mother tree was listed as a key conservation target in the Regulations for the Protection of World Cultural and Natural Heritage in Wuyi Mountain, Fujian Province (Li and Xie, 2014). Wuyishan City Government decided from 2006, Dahongpao mother tree to stop picking, only by the tea professional and technical personnel of Dahongpao mother tree to implement scientific management. Due to the scarcity of the Dahongpao mother tree, most of the Dahongpao tea sold on the market is made from asexually propagated cuttings of the Dahongpao tea tree, and a small portion is made from tea trees bred from Dahongpao tea tree seeds.

Cutting is a method of asexual reproduction of plants, and theoretically, the genome of cuttings is identical to that of the parent and can effectively inherit the characteristics of the parent (Smith et al., 2022). However, plants after asexual reproduction are highly susceptible to environmental influences during growth, which in turn lead to different defense strategies, resulting in differences in growth and quality between them and their parents (Hewitt, 2020; Xiao et al., 2023). Soil is the carrier for plant growth, and the rhizospheric area of direct plant-soil contact, is the area where microorganisms are most active and has a significant impact on plant growth and nutrient acquisition (Dlamini et al., 2022). It has been reported that asexually reproducing plants are more likely to lead to changes in rhizosphere soil microbial diversity and their function under the same environmental conditions, which in turn alters plant growth and quality (Singh, 2021). Lin et al. (2024) found that rhizosphere soil enzyme activities were significantly reduced in asexually propagated sugarcane, and soil microbial biomass nitrogen and beneficial microbial abundance were significantly reduced at both phylum and genus levels. Wang et al. (2024) found that the number of probiotic microorganisms in *Gastrodia* rhizosphere soil gradually decreased after multiple generations of asexual reproduction, while the number of pathogenic bacteria continued to increase, slowing *Gastrodia*’s growth. It is clear that asexually propagated plants do affect soil microbial diversity and its function during growth, which in turn may affect plant growth. Currently, the main propagation method of tea tree is asexual propagation using cuttings. Ng et al. (2018) found that Dahongpao tea tree cuttings planted in the same area had phenotypic traits that were essentially the same as those of the parent tea tree, but with some changes in quality, and hypothesized that this phenomenon may be caused by differences in the effects of cuttings and the parent on soil microorganisms. Do cuttings of tea tree have an effect on rhizosphere soil microbial diversity, and is this effect somewhat different from that of the parent? Little research has been reported in this area, and it is of great significance to reveal this mechanism for tea tree breeding, planting and cultivation management.

Accordingly, in this study, the rhizosphere soil was collected from Dahongpao mother tree (MD) and the first asexually propagated cuttings of Dahongpao (PD) from the same area in the 1980s. Basic chemical indexes and soil enzyme activities were measured in the rhizosphere soils of MD and PD to preliminarily analyze whether they differed in these traits. High-throughput sequencing was further used to analyze the diversity of soil microorganisms and to predict microbial functions, and the analysis was used to obtain the characteristic bacteria and fungi and their potential functions that differed significantly in rhizosphere soils of MD and PD. On this basis, characteristic bacteria and fungi of MD and PD and their relationship with soil nutrients and enzyme activities were analyzed in depth, with a view to providing some references for the cultivation and breeding of tea trees.

## 2 Materials and methods

### 2.1 Soil sampling

The experimental sampling site was located in Jiulongke Scenic Area, Wuyishan City, Fujian Province, China (117°57′19.098″ E, 27°40′17.8212″ N). The objections of this study were Dahongpao mother tree (MD) and the first asexually propagated cutting Dahongpao (PD) in the same area in the 1980’s. The tea tree varieties are all Dahongpao, of which MD is about 390 years old and PD is about 40 years old. MD and PD were planted in the same area, with a straight-line distance of about 20 m between them. In May 2023, rhizosphere soils of MD and PD were collected with three independent replicates of each sample for the determination of high-throughput sequencing, soil chemical indexes, soil enzyme activities, and soil bacteria and fungi. The rhizosphere soil sampling method of tea tree was briefly described as randomly selecting two MD or PD tea trees respectively, removing the residues on the soil surface, shoveling the surface soil layer by layer for about 20 cm, and collecting the soil attached to the root, which is the rhizosphere soil of tea tree (Jia et al., 2024). The rhizosphere soil of 2 tea trees was mixed to make a replicate, totaling about 100 g. Three replicates were taken for each sample.

### 2.2 Determination of soil basic chemical indexes and soil enzyme activities

The methods for the determination of soil available nitrogen, phosphorus, potassium and organic matter content in this study were referred to Ye et al. (2023). Available nitrogen was extracted by NaOH and determined by hydrochloric acid titration. Available phosphorus was extracted by NaHCO_3_ and determined by molybdenum antimony resistance colorimetry. Available potassium was extracted by CH_3_COONH_4_ and determined by flame photometer method. Organic matter content was determined by high-temperature oxidation of potassium dichromate and concentrated sulphuric acid, and titration method of FeSO_4_ solution. Soil urease, protease, acid phosphatase and sucrase activities in this study were determined by the method of Wang et al. (2023), using the Enzyme Linked Immunosorbent Assay Kit (Shanghai Preferred Biotechnology Co., Ltd.). 0.5 g of fresh soil was taken and extracted according to the kit instructions, and then the absorbance was measured using a multiscan spectrum (BioTek Synergy2 Gene 5, Vermont, USA). The urease, protease, acid phosphatase and sucrase activities were measured at 630, 680, 660 and 540 nm, respectively.

### 2.3 High-throughput sequencing analysis of soil bacteria and fungi

Tea tree rhizosphere soil about 1 g was used to extract DNA from soil bacteria and fungi and analyzed by high throughput sequencing. Total soil DNA was extracted using the Bio-Fast DNA extraction kit (BioFlux, Hangzhou, China). Bacterial 16S rDNA amplification was performed using primers 338 F and 806R with ACTCCTACGGGAGGCAGCAG and GGACTACHVGGGTWTCTAAT of sequences, respectively. Fungal ITS rDNA amplification was performed using ITS1 and ITS2 primers with CTTGGTCATTTAGAGGAAGTAA and TGCGTTCTTCATCGATGC of sequences, respectively. The PCR reaction system for both bacteria and fungi was 25 μL, including 2xTaq Plus Master Mix, BSA (2 ng/μL), forward primer (5 μM), reverse primer (5 μM), DNA (30 ng), and dd H_2_O in 12.5, 3.0, 1.0, 1.0, 2.0, and 5.5 μL, respectively. The PCR program for bacterial amplification was set at 95°C for 30 s, 55°C for 30 s, and 72°C for 45 s for 30 cycles. The PCR program for fungi was set to 94°C for 30 s, 52°C for 30 s, 72°C for 30 s, and 35 cycles. Bacterial and fungal PCR products obtained were purified using the Agencourt AMPure XP (Beckman Coulter, Inc., USA) nucleic acid purification kit according to the instruction manual of the kit, and then the library was constructed using the NEB Next Ultra II DNA Library Prep Kit (New England Biolabs, Inc., USA). After library construction was completed, an Agilent 2100 Bioanalyzer (Agilent Technologies, Inc., USA) was used to detect the size of library fragments. Wuhan Maiwei Metabolic Biotechnology Co., Ltd. (Wuhan, China) was commissioned to apply the nanopore sequencing method of Oxford Nanopore sequencing platform for high-throughput sequencing of bacteria and fungi.

The sequenced Fastq data were analyzed using Trimmomatic software (v0.36) and Pear software (v0.9.6), and sequences with quality scores lower than 20, shorter than 120 bp, and containing ambiguous base N were removed (Zhang et al., 2014). Then, the uchime method was used to compare to remove chimeras and non-compliant short sequences of Fasta sequences to obtain clean tags (Rognes et al., 2016). The obtained clean tags were used to cluster the OTUs of the sequences using the uparse algorithm of the Vsearch software (v2.7.1) with the sequence similarity threshold set at 97% (Edgar, 2013). The obtained bacterial and fungal OTU sequences were aligned with the Silva (Release 138) and Unite databases (Release 8.2) using the BLAST algorithm, respectively, to obtain the taxonomic information of the species corresponding to the OTUs. Functions of bacteria and fungi were predicted using FAPROTAX and FUNGuild software.

### 2.4 Quantitative analysis of characteristic bacteria and fungi

Based on the previous studies and analyses, 6 genera of characteristic bacteria (*Candidatus koribacter*, *Pirellula*, *Singulisphaera*, *Sphingomonas*, *Legionella* and *Acidisphaera*) and 4 genera of characteristic fungi (*Penicillium*, *Scopulariopsis*, *Alatospora* and *Tolypocladium*) showed significant differences between MD and PD. Conserved sequences of microorganisms were obtained through NCBI database search and primers were designed (**Supplementary Table S1**) (Jia et al., 2024). The number of strains of 6 genera of characteristic bacteria and 4 genera of characteristic fungi were analyzed by real-time fluorescence quantitative PCR (*q*RT-PCR). Fluorescence quantitative PCR curves were plotted using different concentrations of plasmids, and the copy number of amplified bacteria and fungi in each sample was determined and converted into quantitative data. Briefly, total soil DNA was extracted by the method described previously. The program settings for *q*RT-PCR of the characteristic bacteria were 94°C for 15 s, 60°C 30 s, 72°C 15 s, and 30 cycles; and for the characteristic fungi, 95°C for 45 s, 60°C for 45 s, 72°C for 45 s, and 35 cycles.

### 2.5 Statistical analysis

Rstudio software (version 4.2.3) was used for graphical production and modeling of the data, where the R packages used in box plots, venn diagrams, principal component plots, neutral community models, volcano plots, heat maps, orthogonal partial least squares discrimination analysis (OPLS-DA) model, bubble feature map, technique for order preferenceby similarity to ideal solution (TOPSIS) analysis, metabolic pathway enrichment, redundancy analysis, and PLS-SEM equations using were gghalves 0.1.4, ggVennDiagram 1.5.2, ggbiplot 0.55, minpack.lm 1.2.4, ggplot2 3.5.0, pheatmap 1.0.12, ropls and mixOmics, ggplot2 3.4.0, dplyr 1.1.4, clusterProfiler 4.10.0, vegan 2.6.4, and plspm 0.4.9, respectively (Wickham, 2019).

## 3 Results

### 3.1 Chemical indexes and enzyme activities in rhizosphere soil of tea tree

**Figure 1:**
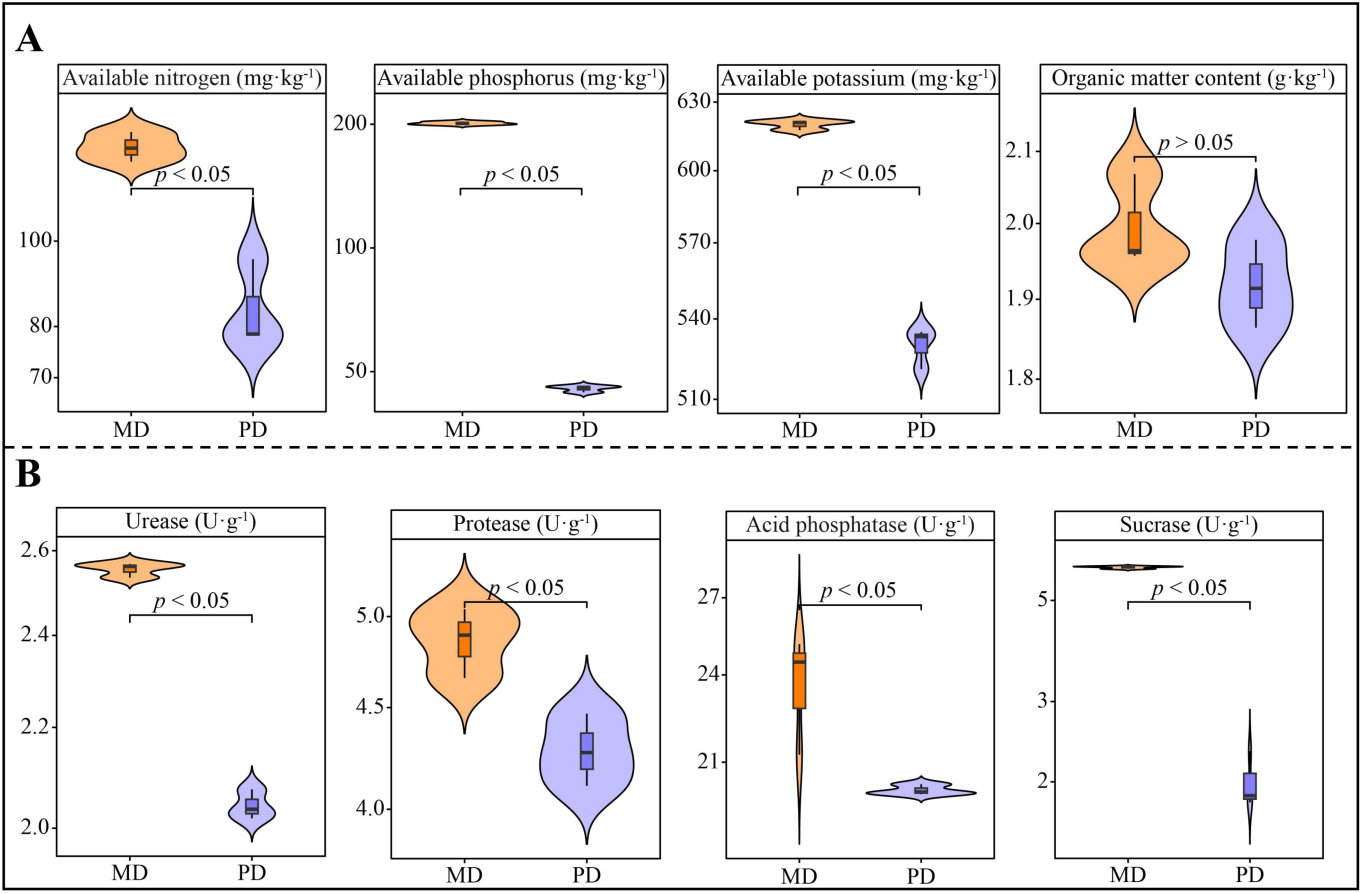
Chemical indexes and soil enzyme activity in rhizosphere soil of tea tree. MD: Dahongpao mother tree; PD: Cutting Dahongpao; **(A)** Analysis of chemical indexes in rhizosphere soil of tea tree; **(B)** Analysis of enzyme activities in rhizosphere soil of tea tree.

Analysis of chemical indexes showed that available nitrogen, available phosphorus, available potassium and organic matter content of rhizosphere soils of tea trees in Dahongpao mother tree (MD) were 127.86 mg·kg^-1^, 201.10 mg·kg^-1^, 620.0 mg·kg^-1^ and 2.00 g·kg^-1^, respectively, while Cutting Dahongpao (PD) was 83.99 mg·kg^-1^, 45.49 mg·kg^-1^, 529.73 mg·kg^-1^ and 1.92 g·kg^-1^ (**Figure 1A**). Among them, available potassium, available phosphorus and available nitrogen differed at significant levels between MD and PD, while organic matter content did not differ significantly. It can be seen that available nutrient content of rhizosphere soil of tea tree in MD was significantly higher than that of PD. Analysis of soil enzyme activities showed that all the enzyme activities of rhizosphere soils of MD were significantly higher than those of PD, as evidenced that the activities of urease, protease, acid phosphatase, and sucrase of MD were 2.55, 4.86, 23.63, and 5.91 U·g^-1^, respectively, while those of PD were 2.04, 4.28, 20.12 and 2.00 U·g^-1^, respectively (**Figure 1B**). It can be seen that the higher rhizosphere soil enzyme activity of MD was more favorable for soil nutrient cycling.

### 3.2 Microbial diversity in rhizosphere soil of tea tree

**Figure 2:**
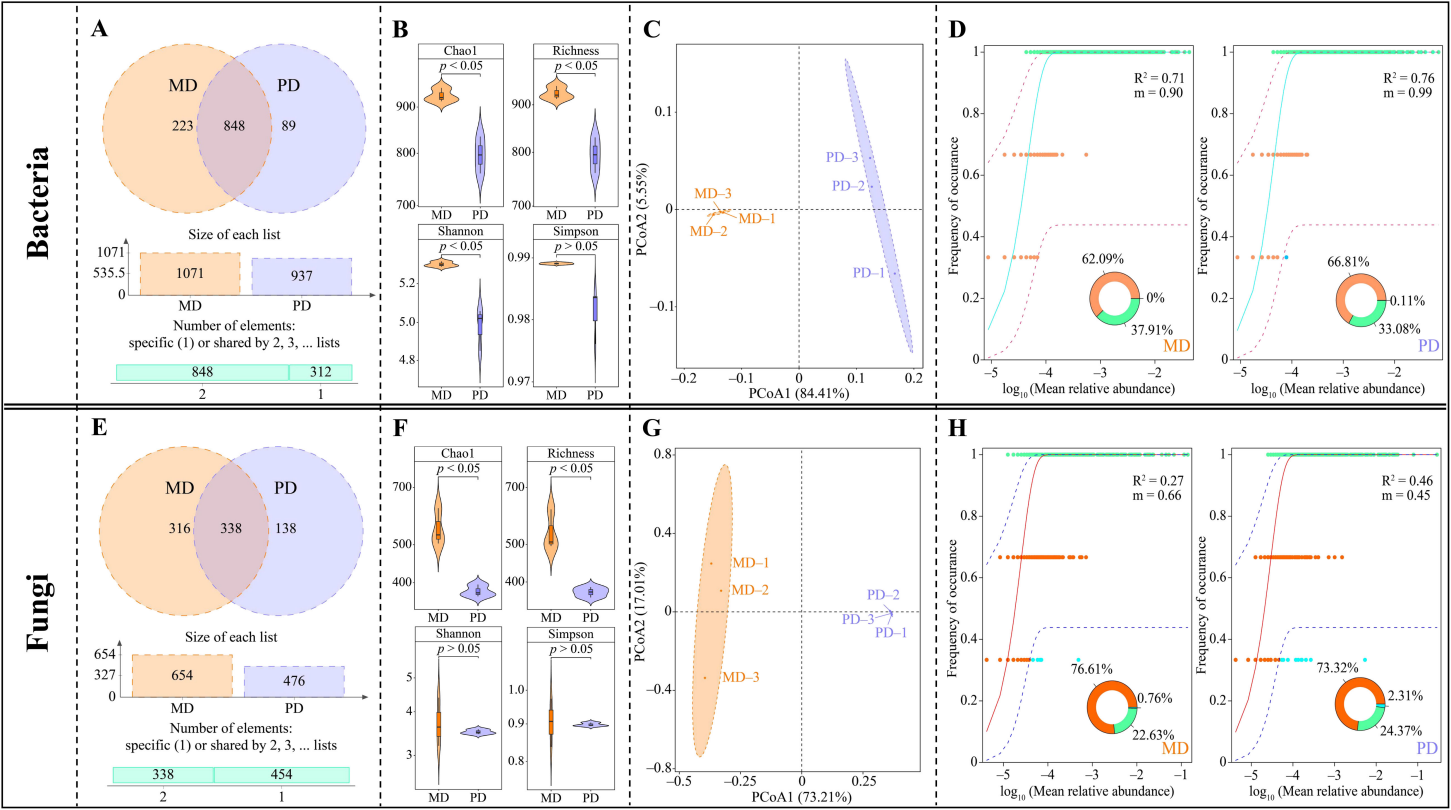
Microbial diversity in rhizosphere soil of tea tree. MD: Dahongpao mother tree; PD: Cutting Dahongpao; **(A)** Venn diagram analysis of bacterial similarity based on OTUs; **(B)** α-diversity index analysis of bacterial communities; **(C)** PCoA analysis of β-diversity of bacterial community; **(D)** neutral community model analysis of bacterial community; **(E)** Venn diagram analysis of fungal similarity based on OTUs; **(F)** α-diversity index analysis of fungal communities; **(G)** PCoA analysis of β-diversity of fungal communities; **(H)** neutral community model analysis of fungal communities.

In this study, the rhizosphere soil of tea trees was investigated, resulting in the detection of a total of 1159 bacterial OTUs. Of these, 848 OTUs were shared between MD and PD, 223 were unique to MD, and 89 were unique to PD (**Figure 2A**). Through the evaluation of α-diversity indexes, it was determined that the Chao1, richness, shannon, and simpson indexes of MD were found to be significantly greater than the equivalent values of PD; the difference of Chao1, richness and shannon indexes between MD and PD reached a significant level (*p* < 0.05) (**Figure 2B**). The results of the PCoA analysis of β-diversity indicated that the two principal components could effectively distinguish between MD and PD, contributing 89.89% of the total variation (**Figure 2C**). It is evident that the difference in bacterial diversity and abundance between MD and PD rhizosphere soils Is significant. Further investigation into the bacterial communities of MD and PD using the neutral community model indicated that MD had a lower R^2^ value and migration rate (m) when compared to PD. Specifically, the R^2^ value and migration rate for MD were 0.71 and 0.90, respectively, while for PD these values were 0.76 and 0.99, respectively (**Figure 2D**). It can be seen that the bacterial community of PD was closer to the neutral model than MD, with a high degree of influence of the bacterial community by stochastic factors, a high degree of dispersal of species, and poor stability of the bacterial community structure.

Fungal sequencing analysis of tea tree rhizosphere soil showed that a total of 792 fungal OTUs were detected in the rhizosphere soil, of which 338 OTUs were identical to MD and PD, 316 were unique to MD, and 138 were unique to PD (**Figure 2E**). The analysis of α-diversity indexes showed that the Chao1, richness, shannon and simpson indexes of MD were higher than those of PD, where the difference between MD and PD in Chao1 and richness indexes reached a significant level (*p* < 0.05), while the difference in shannon and simpson indexes were not significant (*p* > 0.05) (**Figure 2F**). The PCoA analysis of β-diversity showed that the two principal components could effectively differentiate between MD and PD with a total contribution of 90.22% (**Figure 2G**). It can be seen that there was a significant difference in the diversity of fungal communities and their abundance in the rhizosphere soils of MD and PD. Further analysis of the fungal communities of MD and PD using the neutral community model revealed that the R^2^ values of MD and PD were 0.27 and 0.46, respectively, while the migration rates (m) were 0.66 and 0.46, respectively (**Figure 2H**). It can be seen that the fungal community of PD was closer to the neutral model than that of MD, with a high degree of influence by stochastic factors and a relatively low degree of diffusion of species, and a more stable fungal community structure.

### 3.3 Screening of different microorganisms in rhizosphere soil of tea trees

**Figure 3:**
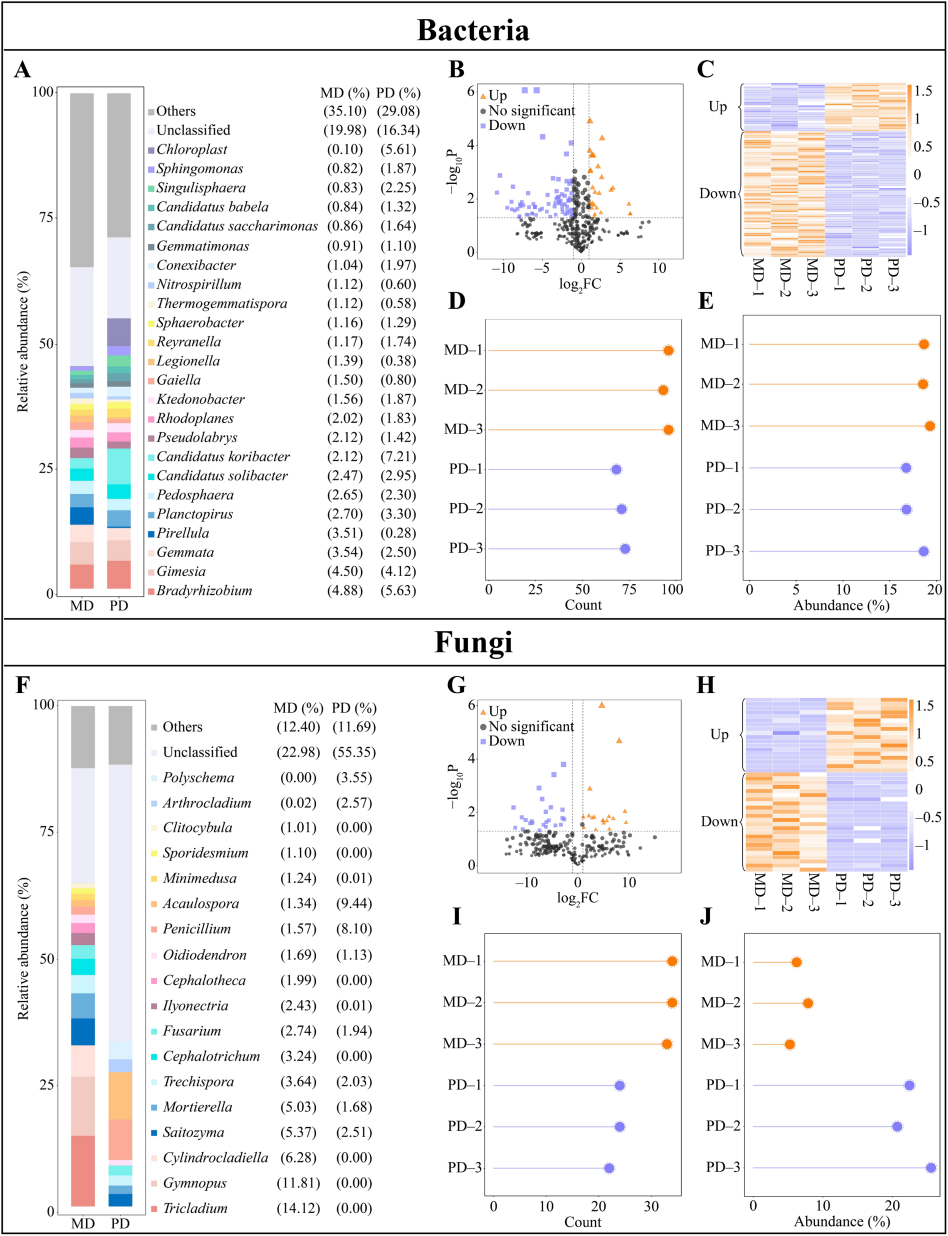
Differential microbial in rhizosphere soil of tea trees. MD: Dahongpao mother tree; PD: Cutting Dahongpao; **(A)** Bacterial genera with abundance greater than 1% in the rhizosphere soil of MD and PD (abundance not greater than 1% is classified as others); **(B)** Volcano plot analysis of differential bacteria; **(C)** Heat map of abundance of differential bacteria; **(D)** Analysis of number of differential bacteria; **(E)** Overall abundance analysis of differential bacteria; **(F)** Fungal genera with abundance greater than 1% in the rhizosphere soil of MD and PD (abundance not greater than 1% is classified as others); **(G)** Volcano plot analysis of differential fungi; **(H)** Heat map of abundance of differential fungi; **(I)** Analysis of number of differential fungi; **(J)** Overall abundance analysis of differential fungi.

Upon conducting the above analysis, this study assessed the abundance of soil microbial communities in the rhizosphere of MD and PD, leading to screen for differential microorganisms. The analysis indicated that there were 18 genera of bacteria with an abundance making up more 1% in MD, led by *Bradyrhizobium* (4.88%), *Gimesia* (4.50%) and *Gemmata* (3.54%). However, PD hosted 19 genera of bacteria with more than 1% abundance, with *Candidatus koribacter* (7.21%), *Bradyrhizobium* (5.63%), and *Chloroplast* (5.51%) represented the highest abundance (**Figure 3A**). Thus, a comprehensive understanding of the rhizosphere soil microbial community between MD and PD was established, indicating that the abundance of different bacteria changed significantly. Exploration of bacterial communities through volcano plots enabled identification of 101 genera that significantly changed in abundance between MD and PD. Specifically, 28 genera were significantly increased and 73 genera were significantly decreased in PD compared with MD (**Figures 3B, C**). Further, an analysis based on the overall numbers of bacterial genera revealed that of 101 bacterial genera that changed significantly, 95 were from MD and 69 were from PD (**Figure 3D**). When considering the overall abundance of these genera, the percentage of abundance of significantly changed bacterial genera in MD was 18.85%, while the percentage of PD was 17.39% (**Figure 3E**). It can be seen that there was a significant difference in the abundance of a large number of bacteria in the rhizosphere of MD and PD.

In addition, the analysis of fungal abundance in the rhizosphere soil of tea tree showed that there were 18 fungal genera with abundance greater than 1% in the rhizosphere soil of MD, and the top 3 genera were *Tricladium* (14.12%), *Gymnopus* (11.81%), and *Cylindrocladiella* (6.28%); 9 genera with abundance greater than 1% were found in the rhizosphere soil of PD, and the top 3 genera were *Acaulospora* (9.44%), *Penicillium* (8.10%), and *Polyschema* (3.55%) (**Figure 3F**). It was seen that there was a significant difference in the abundance of different fungi in rhizosphere soils of MD and PD. Screening of fungi with significant differences between MD and PD using volcano plots revealed that a total of 42 fungal genera had significant differences in abundance between MD and PD, of which 18 genera were significantly increased and 24 genera were significantly decreased in PD compared to MD (**Figures 3G, H**). Second, analyzed in terms of overall numbers, of the 42 fungal genera with significant changes, there were 34 genera in MD and 23 genera in PD; and in terms of overall abundance, the percentage of fungal genera with significant changes in abundance accounted for 6.46% in MD, while the percentage of PD accounted for 22.90% (**Figures 3I, J**). It was seen that there was a significant difference in the abundance of a large number of fungi in rhizosphere soils of MD and PD.

### 3.4 Screening and abundance analysis of characteristic microorganisms in the rhizosphere soil of tea tree

**Figure 4:**
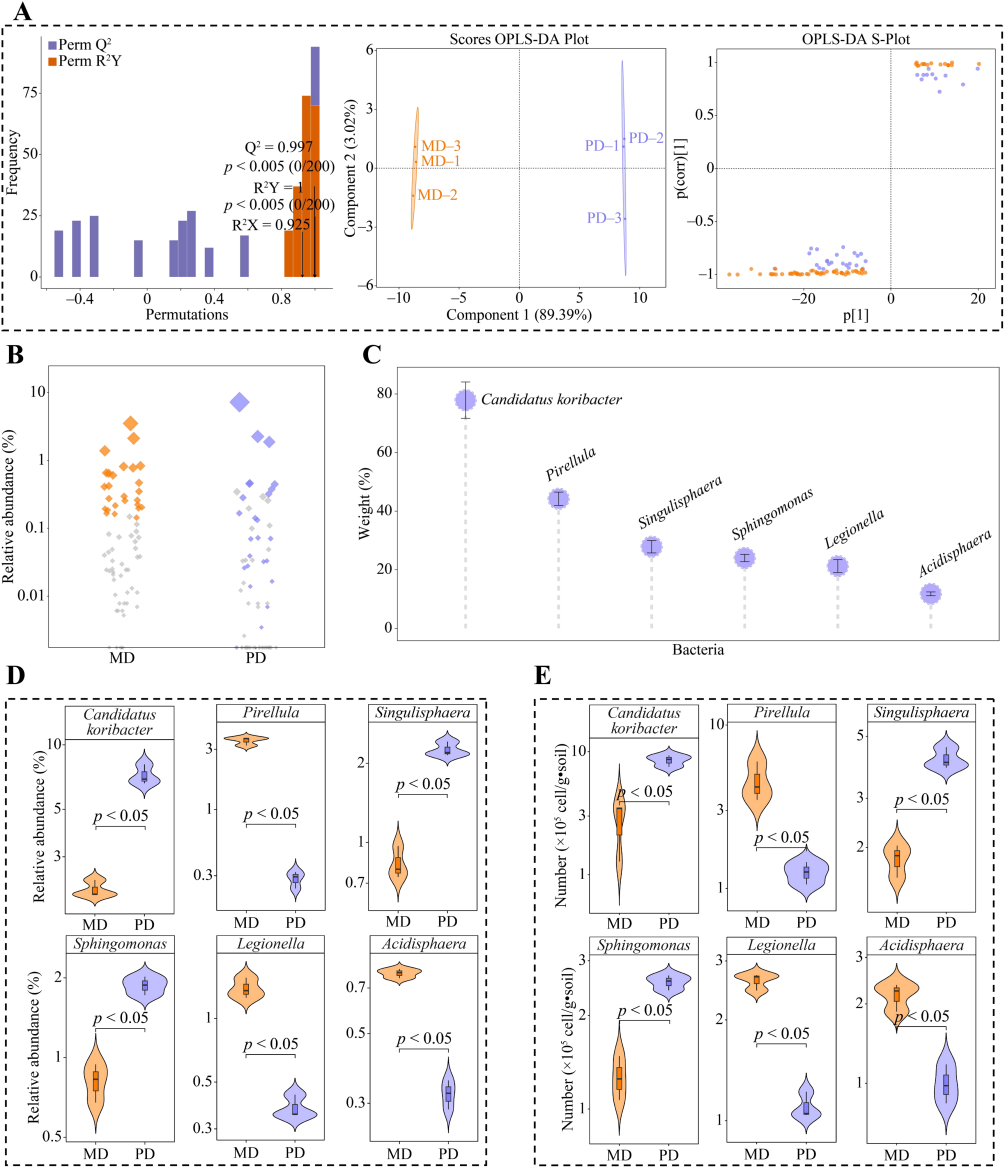
Screening characteristic bacteria and their abundance analysis in rhizosphere soil of tea tree. MD: Dahongpao mother tree; PD: Cutting Dahongpao; **(A)** MD and PD constructed OPLS-DA model to screen for key differential bacteria; **(B)** Bubble feature map to screen for key bacteria with more than 90% abundance; **(C)** TOPSIS analysis of key bacteria to obtain characteristic bacteria that distinguish MD from PD with a weight of 10% or more; **(D)** Analysis of abundance of characteristic bacteria; **(E)** *q*RT-PCR analysis of characteristic bacteria.

Based on the above analysis, 101 genera of bacteria were significantly different in both MD and PD. The OPLS-DA model of MD and PD was further constructed to screen key differential bacteria. The model effectively distinguish MD and PD across different regions, as demonstrated by the model’s fit (R^2^Y) and predictability (Q^2^) (*p* < 0.005). Secondly, the model identified 67 genera of key differential bacteria with importance projection values (VIP) greater than 1 (**Figure 4A**). Using bubble feature maps, 67 genera of key differential bacteria were analyzed and identified. Among these, 26 genera displayed more than 90% abundance (**Figure 4B**). 67 genera of key differential bacteria were thoroughly analyzed and identified, making them key bacterial genera. Moreover, TOPSIS was used to assess the weights of 26 key bacterial genera in distinguishing MD from PD. The assessment showed that 6 bacterial genera had weights of 10% or more, making them characteristic bacteria, namely *Candidatus koribacter*, *Pirellula*, *Singulisphaera*, *Sphingomonas*, *Legionella*, and *Acidisphaera*, (**Figure 4C**). Abundance analysis of characteristic bacteria showed that the abundance of *Candidatus koribacter*, *Singulisphaera* and *Sphingomonas* was significantly greater in PD than in MD, while the abundance of *Pirellula*, *Legionella* and *Acidisphaera* was significantly greater in MD than in PD (**Figure 4D**). Further *q*RT-PCR was used to analyze characteristic bacteria in the rhizosphere soils of MD and PD, which showed that PD had significantly higher counts of *Candidatus koribacter*, *Sphingomonas* and *Sphingomonas* than MD in rhizosphere soils, while *Pirellula*, *Legionella* and *Acidisphaera* had significantly lower counts than MD (**Figure 4E**). This result validates the results of the changes in the abundance of the characteristic bacteria and also indicates that there are significant differences between the bacterial communities of MD and PD, especially the characteristic bacteria.

**Figure 5:**
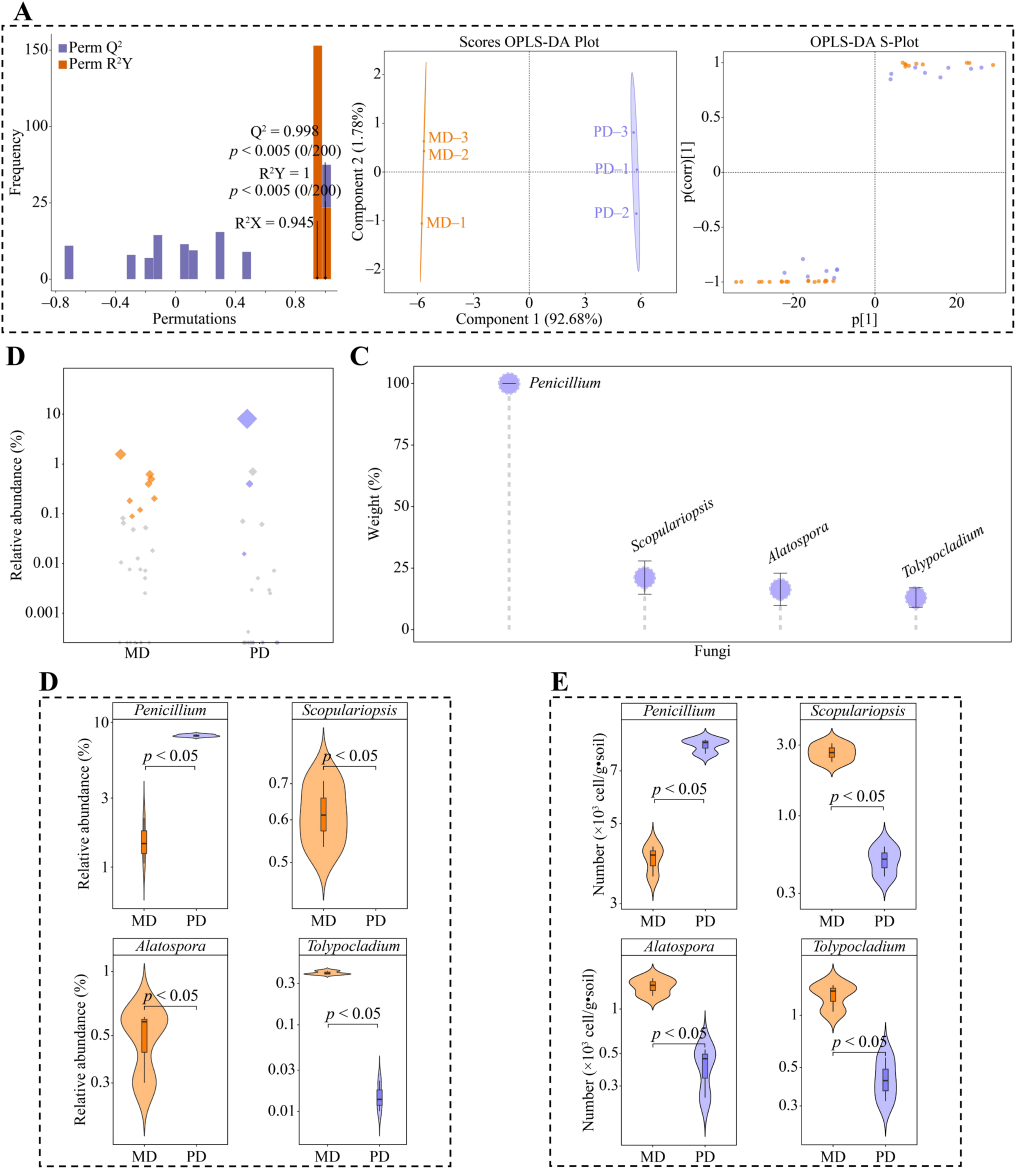
Screening characteristic fungi and their abundance analysis in rhizosphere soil of tea tree. MD: Dahongpao mother tree; PD: Cutting Dahongpao; **(A)** MD and PD constructed OPLS-DA model to screen for key differential fungi; **(B)** Bubble feature map to screen for key fungi with more than 90% abundance; **(C)** TOPSIS analysis of key fungi to obtain characteristic fungi that distinguish MD from PD with a weight of 10% or more; **(D)** Analysis of abundance of characteristic fungi; **(E)** *q*RT-PCR analysis of characteristic fungi.

Moreover, in this study, using the 42 genera of fungi that exhibited considerable differences in MD and PD according to the above analysis, we constructed the OPLS-DA model for MD and PD. This model was used to identify the key differential fungi. The results showed that the OPLS-DA model constructed for MD and PD could effectively distinguish MD and PD in different regions, and the fit (R^2^Y) and predictability (Q^2^) of model, reached significant levels (*p* < 0.005), and 27 genera of key differential fungi with variable importance projection (VIP) values greater than 1 were obtained by the model (**Figure 5A**). Bubble feature map was used to analyze the 27 genera of key differential fungi, and 8 key fungi were obtained with more than 90% abundance (**Figure 5B**). TOPSIS was further used to analyze the weights of 8 genera of key fungi in distinguishing MD from PD, and the results showed that there were 4 genera of characteristic fungi with weights of 10% or more when distinguishing MD from PD, namely *Penicillium*, *Scopulariopsis*, *Alatospora* and *Tolypocladium* (**Figure 5C**). Abundance analysis of characteristic fungi showed that the abundance of *Scopulariopsis*, *Alatospora* and *Tolypocladium* was significantly greater in MD than in PD, while the abundance of *Penicillium* was significantly less in MD than in PD (**Figure 5D**). Further *q*RT-PCR was used to analyze the characteristic fungi of MD and PD rhizosphere soils, which showed that the number of *Scopulariopsis*, Alatospora and *Tolypocladium* was significantly higher in MD rhizosphere soils than PD, while the number of *Penicillium* was significantly lower than PD (**Figure 5E**). This result validated the results of changes in the abundance of characteristic fungi, and also indicated that there were significant differences between the fungal communities of MD and PD, especially the characteristic fungi.

### 3.5 Functional analysis of characteristic microorganisms in the rhizosphere soil of tea tree

**Figure 6:**
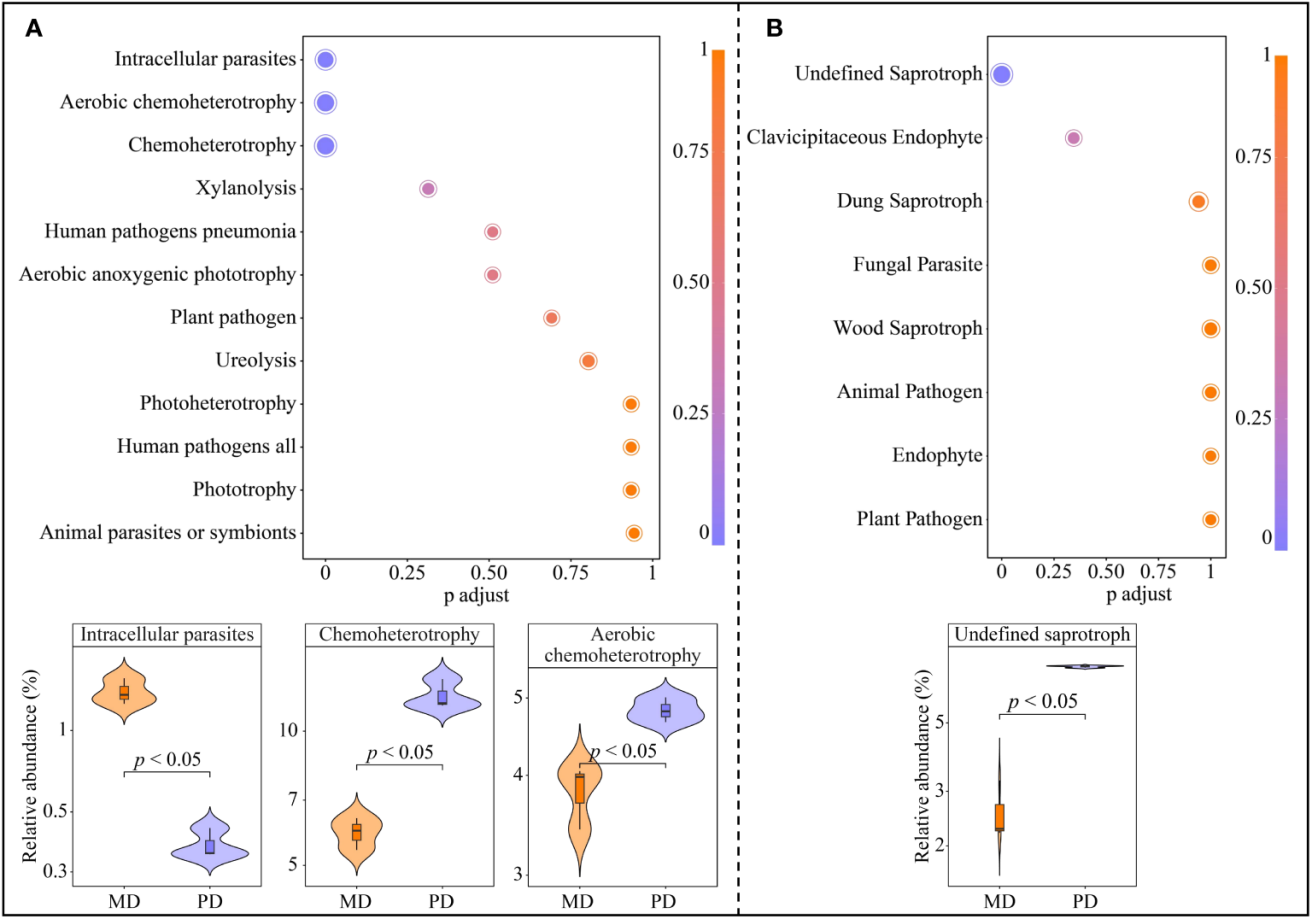
Functional of characteristic bacteria and fungi in the rhizosphere soil of tea tree. MD: Dahongpao mother tree; PD: Cutting Dahongpao; **(A)** Enrichment of metabolic pathways to characteristic bacterial functions and their intensity analysis; **(B)** Enrichment of metabolic pathways to characteristic fungal functions and their intensity analysis.

The above analysis obtained 6 characteristic bacteria from a total of 43 OTUs, further metabolic pathway enrichment was performed based on functions corresponding to each OUTs. A total of 12 metabolic pathways were enriched, of which 3 metabolic pathways were significantly enriched, namely intracellular parasites, aerobic chemoheterotrophy and chemoheterotrophy (**Figure 6A**). The abundance of intracellular parasites pathway was significantly greater in MD than in PD, whereas the abundance of aerobic chemoheterotrophy and chemoheterotrophy pathways was significantly less in MD than in PD. In addition, the above analysis obtained 4 characteristic fungi from 61 OTUs, further metabolic pathway enrichment was performed based on functions corresponding to each OUTs. A total of 8 metabolic pathways were enriched, of which only 1 metabolic pathway was significantly enriched, namely undefined saprotroph (**Figure 6B**). The abundance of undefined saprotroph pathway was significantly greater in PD than in MD. It can be seen that the differences in the abundance of characteristic microorganisms in the rhizosphere soils of MD and PD led to some differences in the function.

### 3.6 Interaction effects between rhizosphere soil microorganisms and different indexes of tea tree

**Figure 7:**
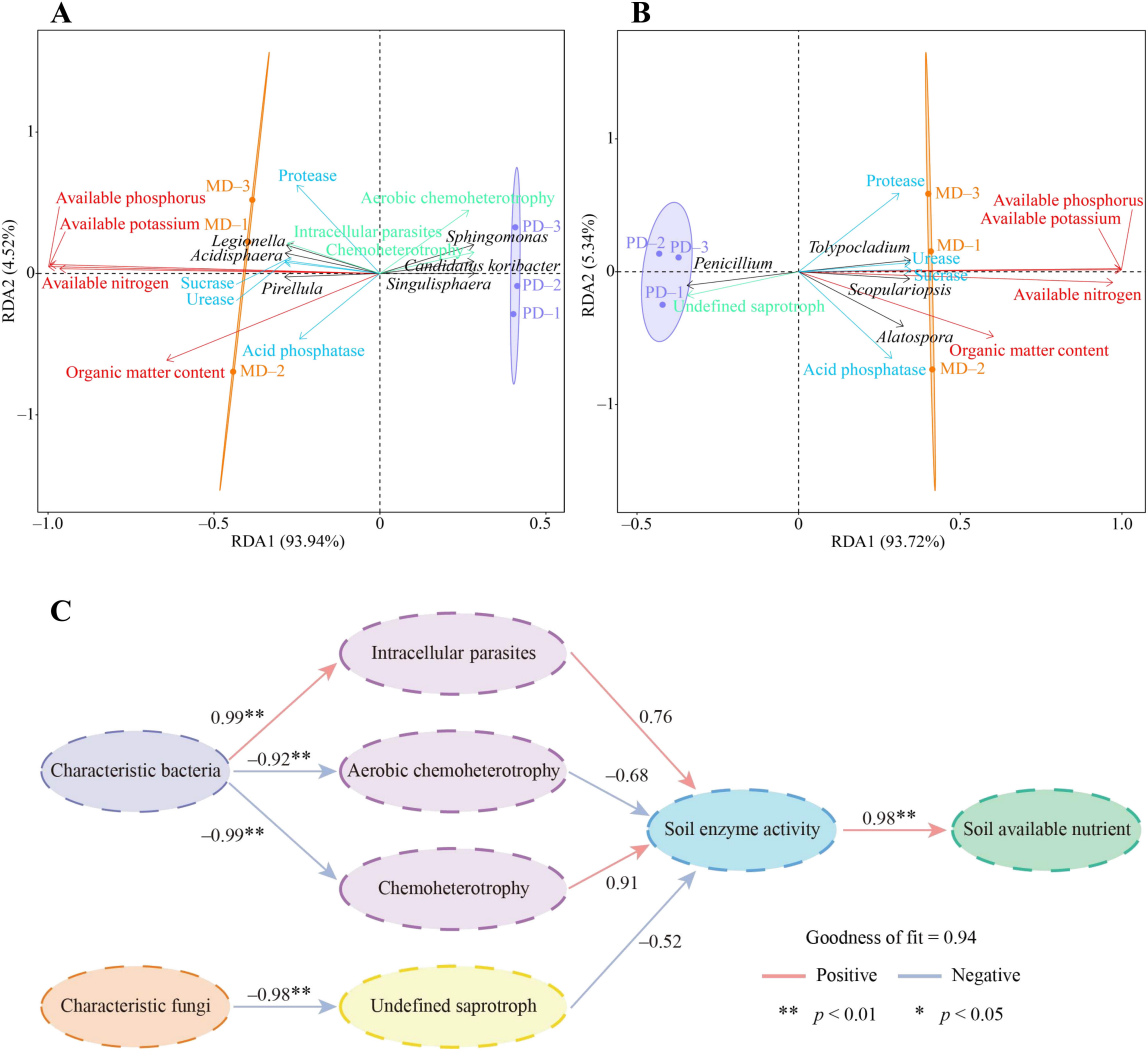
Interaction effects of characteristic microorganisms and functions in rhizosphere soil of tea tree with soil chemical indexes and soil enzyme activities. MD: Dahongpao mother tree; PD: Cutting Dahongpao; **(A)** Redundancy analysis of characteristic bacteria and their functions with soil chemical indexes, and soil enzyme activities; **(B)** Redundancy analysis of characteristic fungi and their functions with soil chemical indexes, and soil enzyme activities; **(C)** Construction of PLS-SEM equations between different indexes. .

In this study, redundancy analysis and PLS-SEM equations were further used to analyze the interaction effects between rhizosphere soil microorganisms and different indexes of tea tree. Redundancy analysis showed that available nitrogen, available phosphorus, available potassium, and organic matter contents of the soil were significantly correlated with 3 characteristic bacteria, including *Pirellula*, *Legionella*, and *Acidisphaera*, and with the intensity of intracellular parasites pathway, as well as with the activity of urease, protease, acid phosphatase, and sucrase in the soil (**Figure 7A**). While 3 characteristic bacteria such as *Candidatus koribacter*, *Sphingomonas*, and *Sphingomonas* were significantly correlated with the intensity of metabolic pathways such as aerobic chemoheterotrophy, chemoheterotrophy. Secondly, it was found that available nitrogen, available phosphorus, available potassium, and organic matter contents of the soil were significantly correlated with 3 characteristic fungi such as *Scopulariopsis*, *Alatospora*, and *Tolypocladium*, and significantly correlated with the activity of urease, protease, acid phosphatase, and sucrase in the soil (**Figure 7B**). While *Penicillium* of characteristic fungi was significantly correlated with the intensity of undefined saprotroph metabolic pathway.

In addition, this study further constructed PLS-SEM equations between different indexes to analyze their interaction effects, and the results showed that the effect of characteristic bacteria was positive on intracellular parasites pathway, and negative on aerobic chemoheterotrophy and chemoheterotrophy pathways, and both of them reached the significant level (**Figure 7C**). Intracellular parasites pathway had a positive effect on soil enzyme activity while aerobic chemoheterotrophy and chemoheterotrophy pathways had a negative effect on soil enzyme activity. Soil enzyme activity had a positive effect on soil available nutrient content at a significant level. Secondly, it was found that characteristic fungi had a significant negative effect on undefined saprotroph pathway, undefined saprotroph pathway had a negative effect on soil enzyme activity, and soil enzyme activity had a significant positive effect on soil available nutrient content (**Figure 7C**). It can be seen that changes in soil characteristic microorganisms affected soil microbial function, which in turn altered soil enzyme activities and affected available nutrient content of the soil.

## 4 Discussion

Plants can’t grow healthily without a supply of nutrients, and soil is an important place for plants to get them. The nutrient content of the soil and its transformation capacity directly affects the ability of plants to absorb nutrients (Soares et al., 2019). Soil enzyme activity is an important index for evaluating soil nutrient transformation capacity, and its activity level directly affects soil available nutrient content (Zielewicz et al., 2021; Xu et al., 2020). In the study, it was found that urease, protease, acid phosphatase and sucrase activities were significantly higher in rhizosphere soils of tea tree in MD than in PD. The available potassium, available phosphorus and available nitrogen contents of the rhizosphere soils of MD were also significantly greater than those of PD. It has been reported that soil available nutrient content is significantly and positively correlated with plant growth, and increasing the available nutrient content of the rhizosphere soil of plants is favorable for nutrient uptake by the plant root system, which in turn promotes the growth of plants (Tian et al., 2020). It can be seen that compared with PD, MD is more conducive to enhancing the activity of rhizosphere soil nutrient cycling-related enzymes, more conducive to improving the nutrient cycling capacity of the soil and increasing the available nutrient content of the rhizosphere soil, which in turn promotes the growth of tea tree.

The plant rhizosphere is a complex micro-ecological environment, and changes in the community structure of rhizosphere microorganisms and their functions affect soil nutrient transformation and nutrient uptake by the plant root system (Zhong et al., 2020; Liu et al., 2022). In the study, significant differences were found in the bacterial and fungal community structure of the rhizosphere soils of tea tree in MD and PD, as evidenced by the fact that the diversity of both bacterial and fungal communities was higher in MD than in PD. Second, compared with MD, the bacterial and fungal communities in PD were highly influenced by stochastic factors, and the bacterial community had a high degree of species dispersal and a poorly stabilized community structure, whereas the fungal community had a relatively low degree of species dispersal and a more stable community structure. It has been reported that deterioration of plant-grown soils usually leads to a shift in the microbial community structure of the rhizosphere soil, with a decrease in microbial diversity, and the soil microorganisms begin to shift from bacterial to fungal types (Liu et al., 2019; Hang et al., 2020). The number of beneficial microorganisms in the soil decreases and the number of pathogenic microorganisms increases, which in turn leads to soil texture in the direction of deterioration, affects nutrient uptake by the plant root system, and hinders plant growth (Yang et al., 2020). It can be seen that the microbial community structure of the rhizosphere soil of PD is more single and highly susceptible to environmental factors compared to MD, and this phenomenon may lead to changes in the function of its soil microorganisms. Therefore, the study further explored the characteristic microorganisms and their functions that were significantly different in MD and PD, and it was found that there were 6 genera of characteristic bacteria that were significantly different in MD and PD, among which, the abundance of *Candidatus koribacter*, *Singulisphaera*, and *Sphingomonas* was significantly greater in PD than in MD, whereas the abundance of *Pirellula*, *Legionella*, and *Acidisphaera* was significantly less in PD than in MD. *Candidatus koribacter* and *Legionella* have been reported to be common soil pathogens with inhibitory effects on plant growth (Liu et al., 2021; Casati et al., 2009). The abundance of *Singulisphaera* increases under environmental stresses, especially when the soil is deficient in phosphorus and potassium, which highly promotes *Singulisphaera* reproduction (Zuo et al., 2022). Environmental stresses also tend to induce an increase in *Sphingomonas* abundance with the aim of alleviating stress and promoting plant growth (Asaf et al., 2020). *Pirellula* is associated with nitrogen cycling in soil and it promotes soil nitrogen transformation and increases available nitrogen content (Ravinath et al., 2022). *Acidisphaera* increases soil phosphatase activity, which in turn promotes soil phosphorus cycling and increases soil available phosphorus content (Keet et al., 2021). As can be seen, from the bacterial point of view, pathogenic characteristic bacteria were present in the rhizosphere soils of both MD and PD, however, the abundance of *Pirellula* and *Acidisphaera* associated with soil nutrient cycling was significantly higher in the rhizosphere soils of MD than that of PD, whereas the abundance of *Singulisphaera* and *Sphingomonas* associated with environmental stresses was significantly lower than that of PD. Second, this study also found that the functions of the characteristic bacteria were mainly enriched in 3 metabolic pathways, where the abundance of intracellular parasites pathway was significantly greater in MD than in PD, whereas the abundance of aerobic chemoheterotrophy and chemoheterotrophy pathways was significantly less in MD than in PD. Intracellular parasites pathway had a positive effect on soil enzyme activity while aerobic chemoheterotrophy and chemoheterotrophy pathways had a negative effect on soil enzyme activity, and soil enzyme activity had a positive effect on soil available nutrient content at a significant level. It can be seen that compared with PD, the soil texture of MD is relatively better, and its soil nutrient cycling-related enzyme activities are stronger, which is more conducive to soil nutrient cycling and increases the available nutrient content of the soil.

In addition, 4 genera of characteristic fungi were found to be significantly different between MD and PD in this study, among which, the abundance of *Scopulariopsis*, *Alatospora* and *Tolypocladium* was significantly greater in MD than in PD, whereas the abundance of *Penicillium* was significantly less in MD than in PD. It has been reported that *Scopulariopsis*, *Tolypocladium* and *Penicillium* are beneficial bacteria that secrete antibiotics and can effectively inhibit soil pathogens and promote plant growth (Cui et al., 2022; Brückner and Degenkolb, 2020; Prudnikova et al., 2021). *Alatospora* not only inhibits pathogenic fungi, but it also enhances soil sucrase and acid phosphatase activities and increases soil available nitrogen and phosphorus contents (Wang et al., 2022). It can be seen that from the fungal point of view, characteristic fungi in the rhizosphere soil of both MD and PD have the function of strongly inhibited pathogenic bacteria, however, MD is relatively stronger in suppressing pathogens compared to PD. The study also revealed that the functions of the characteristic fungi were mainly enriched in undefined saprotroph pathway and the abundance of undefined saprotroph pathway was significantly greater in PD than in MD. Characteristic fungi showed a significant negative effect on undefined saprotroph metabolic pathway, undefined saprotroph metabolic pathway showed a negative effect on soil enzyme activity, and soil enzyme activity showed a significant positive effect on soil available nutrient content. It can be seen that the rhizosphere soil of PD tea trees is more susceptible to decay compared to MD, which in turn inhibits soil enzyme activities and reduces soil effective nutrient content.

The study’s overall aim was to scrutinize the diversity and functional changes of bacteria and fungi in the rhizosphere soils of tea trees in MD and PD and their effects on the transformation of soil nutrients. It was found that 6 genera of characteristic bacteria and 4 genera of characteristic fungi were significantly different between MD and PD rhizosphere soils. Rhizosphere soil available nutrient content and soil enzyme activity were significantly higher in MD than in PD, mainly originating from the contribution of *Pirellula* and *Acidisphaera* of characteristic bacteria and *Alatospora* of characteristic fungi. Secondly, MD had a stronger ability to inhibit soil pathogens than PD, mainly derived from the contribution of *Scopulariopsis* and *Tolypocladium* of characteristic fungi. In conclusion, compared with PD, the soil texture of MD was relatively better, and its soil nutrient cycling-related enzyme activities were stronger, which was more conducive to soil nutrient cycling and increased the available nutrient content of the soil, which in turn promoted the growth of tea trees. This study revealed the rhizosphere soil microbial diversity and its functional differences between Dahongpao mother tree and cutting Dahongpao, indicating that there were significant differences in rhizosphere soil microbial functions between the tea tree cuttings and the mother tree, and that the mother tree was better adapted to the environment than its cuttings. This study provides an important reference for the cultivation and management of tea tree cuttings and microbial regulation of tea tree growth.

## Data Availability

The datasets presented in this study can be found in online repositories. The names of the repository/repositories and accession number(s) can be found in the article/**Supplementary Material**.
