# Supplementary material for: Analysis of rhizosphere soil microbial diversity and its functions between Dahongpao mother tree and cutting Dahongpao
Source: Front Plant Sci. 2024 Sep 6;15:1444436. doi: 10.3389/fpls.2024.1444436 (PMC11412831; doi:10.3389/fpls.2024.1444436)
Supplement: Supplementary file 1 [file DataSheet1.pdf]

Supplementary data

Table S1 *q*RT-PCR primers for characteristic bacteria and fungi

No.	Microorganism	Forward primer	Reverse primer	Product (bp)
1	*Candidatus koribacter*	GAATTCCCGGTGTAGCGGTG	AGCGTCAGTTGTGGTCCAG	84
2	*Pirellula*	TGTAAACCGCTGTCGTAGGG	CCTTCCTCCTAGGTTCAGTCA	72
3	*Singulisphaera*	AACCGTGCGAACGTTATTCG	GATTTCAGCGACAGACGCAC	75
4	*Sphingomonas*	ACATGCAAGTCGAACGGGTA	TCCCGCTGTTATTCCGAACC	92
5	*Legionella*	AACACATGCAAGTCGAACGG	TTCCTACGCGTTACTCACCC	81
6	*Acidisphaera*	ATTGGACAATGGGCGAAAGC	GGGTACCGTCATCATCGTCC	100
7	*Penicillium*	CCAACCTCCCACCCATGTTT	GAGTTCTAGGTGTCTCCGGC	110
8	*Scopulariopsis*	AAGCGTACTGCACGTTCTGA	GCATTTCGCTGCGTTCTTCA	97
9	*Alatospora*	AAAGTCGTAACAAGGTTTCCGTAG	AGTAGGCCGAAGCCTCTAA	72
10	*Tolypocladium*	AACCAGCGGAGGGATCATTA	GCCGAGGCAACAGCAAA	77
